# Quantitative catalogue of mammalian mitotic chromosome-associated RNAs

**DOI:** 10.1038/s41597-023-02884-8

**Published:** 2024-01-06

**Authors:** Le Zhang, Chuansheng Hu, Zeqian Xu, Hua Li, Bishan Ye, Xinhui Li, Daniel M. Czajkowsky, Zhifeng Shao

**Affiliations:** State Key Laboratory of Systems Medicine for Cancer, School of Biomedical Engineering, Shanghai Jiao Tong University, Shanghai, 200240 China

**Keywords:** Chromosomes, Chromatin

## Abstract

The faithful transmission of a cell’s identity and functionality to its daughters during mitosis requires the proper assembly of mitotic chromosomes from interphase chromatin in a process that involves significant changes in the genome-bound material, including the RNA. However, our understanding of the RNA that is associated with the mitotic chromosome is presently limited. Here, we present complete and quantitative characterizations of the full-length mitotic chromosome-associated RNAs (mCARs) for 3 human cell lines, a monkey cell line, and a mouse cell line derived from high-depth RNA sequencing (3 replicates, 47 M mapped read pairs for each replicate). Overall, we identify, on average, more than 20,400 mCAR species per cell-type (including isoforms), more than 5,200 of which are enriched on the chromosome. Notably, overall, more than 2,700 of these mCARs were previously unknown, which thus also expands the annotated genome of these species. We anticipate that these datasets will provide an essential resource for future studies to better understand the functioning of mCARs on the mitotic chromosome and in the cell.

## Background & Summary

One of the most dramatic molecular-level changes that occurs during the cell cycle is the formation of highly compact mitotic chromosomes from the interphase chromatin^1^. This is critical for the faithful transmission to the daughter cells of not only the genomic material but also the other constituents that are tightly bound to the chromosomes, including both proteins and RNA^2–4^. With regards to the former, there have been significant recent advances in our understanding of the composition^5^ and functioning of the proteins associated with the mitotic chromosome^6^, including as molecular “bookmarks” that are thought to be necessary to maintain cellular phenotype^7^. Yet, by contrast, we presently know much less about the RNA composition of mitotic chromosomes, and even less about their functioning. This is despite long-established evidence that a significant fraction of the mitotic chromosome mass is owing to the RNA components^2^. Since it has been demonstrated that there are many RNA molecules that are associated with interphase chromatin that play critical regulatory roles in many genomic processes^8–11^, and also several examples of specific RNA species that perform important functions when associated with the mitotic chromosome^12,13^, it is expected that there are many mitotic chromosome-associated RNAs (mCARs) that remain to be identified, whose characterization will prove to be essential for an understanding of the formation and functioning of the mitotic chromosome in the cell.

With this expectation in mind, there have been some attempts in the last few years to describe the repertoire of mCARs. In particular, using a targeted method that is based on 5’-tag sequencing, our group provided the first systems-wide description of mCAR species in any mammalian cells (mouse 3T3 cells)^14^. This work identified more than one thousand mCARs, most of which are non-coding RNAs (ncRNAs). However, as this was based on 5’-tag sequencing, it was not possible to identify the full-length versions of these mCAR transcripts. In addition, it was also not possible to determine the degree of conservation of these mCARs with other cell types or between different species. Importantly, while this work identified the RNAs that were associated with the chromosome, the precise extent of enrichment could not be determined, owing to the limitations of the methodology employed. Thus, whether the association of any particular mCAR with the chromosome was simply a consequence of an overall high abundance of this species in the cell, or rather the mCAR was specifically enriched on the chromosome could not be resolved. For mCAR species of the former type, their association to the chromosome might reflect a means by which the daughter cells effectively obtains the most abundant RNA species of the mother cell, whereas for the latter type, these mCAR species might be expected to play critical roles either in the assembly/disassembly of the mitotic chromosome or perhaps in the maintenance of cellular identity (akin to the aforementioned molecular “bookmarking” function of specific proteins associated with the mitotic chromosome). Recently, Shen *et al*. sequenced the RNA extracted from mitotic pellets of two human cell lines and mapped the sequenced reads to a large number of annotated genes ( >13,000 per cell)^15^. But despite this high number, no novel genes were noted, perhaps owing to a limited sequencing depth. Moreover, similar to our previous approach, mitotic enrichments of these mCARs could not be precisely assessed owing to limitations in the methodology utilized.

Here, using highly purified, intact mammalian mitotic chromosomes under conditions that maintain the structure, morphology, and components of the native chromosomes, we present a comprehensive characterization of the mCARs for 5 different mammalian cells with a high sequencing depth (3 replicates, 47 M mapped read pairs for each replicate, on average). We characterize 3 epithelial human cells (ARPE-19, A549, HT-1080), 1 mouse fibroblast cell (STO) and 1 monkey fibroblast cell (CV-1 derived from the African green monkey, *Cercopithecus aethiops*) to enable comparisons between both different cell types as well as mammalian species. Moreover, using genomic DNA as the normalizer between the cytosolic fraction and the mitotic fraction, we are able to determine the absolute level of enrichment for each mCAR species, thus providing the first systems-wide quantitative characterization of mCARs in any cell as well.

Overall, we found on average 20,443 transcripts (ranging from 7,147 to 40,644) per cell-type, including 19,891 full-length annotated mCAR transcripts and 552 novel RNAs (including isoforms). Of these mCARs, we show that more than 5,200 are enriched on the mitotic chromosome ( >1.5-fold higher than in the cytosol) (Table S1-S2 in figshare^16^). In terms of overall abundance, the majority of the mCARs are ncRNAs, particularly snoRNAs. However, in terms of specific mCAR species, there are only ~5,000 different ncRNA species but ~13,000 mRNA transcripts on average per cell-type, albeit most of the latter exhibit low copy numbers, consistent with previous findings^17^. Within the human cells, we find that there are 5,731 mCARs that are shared between these cells, suggesting a considerable degree of conserved functionality of the mCARs. Yet, in terms of enriched mCARs ( >1.5-fold higher than the cytosolic fraction), there are only 821 mCARs that are shared between the three human cells, indicating that there is also substantial cell-type specificity in the mCARs, which possibly contributes to the maintenance of cell identity. Taking advantage of our high sequencing depth, we also identified over 100 transcripts that map to genes that were not previously annotated in the human and mouse genomes, and over 2,000 transcripts that map to novel genes in the less annotated green monkey genome. Thus, our results also expand the annotation of the genomes for each of these mammalian species. Hence, with these datasets, we provide the most comprehensive and quantitative catalogue of mCAR species to date, which we anticipate will find use in future bioinformatic and molecular biological studies designed to better understand the functioning of these specific mCARs on the mitotic chromosome and in the cell.

## Methods

**Fig. 1:**
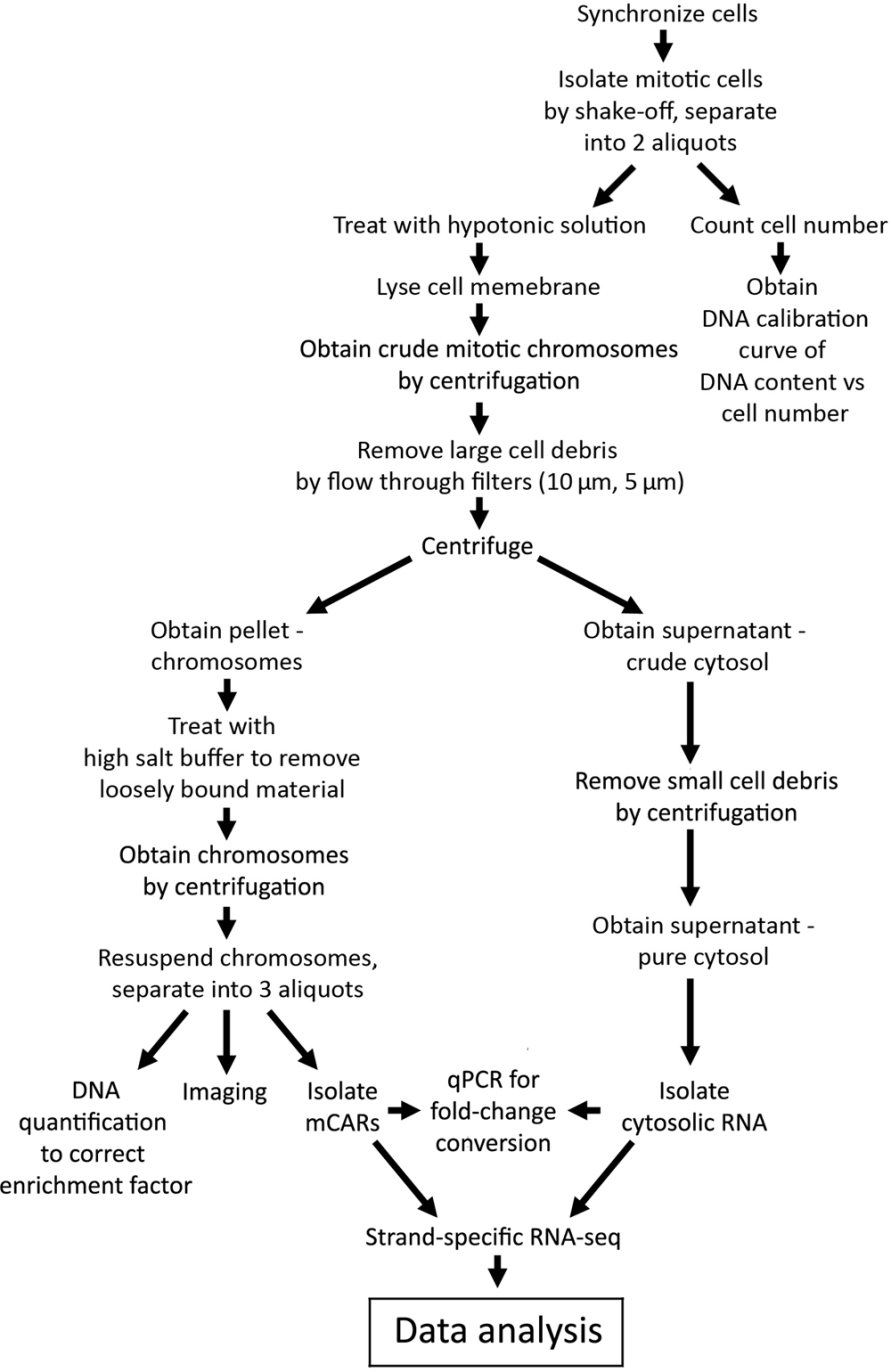
**Schematic overview of the overall experimental workflow**. Of particular note is the determination of the DNA calibration curve from an initial aliquot of mitotic cells which is used to normalize the reads obtained from both the cytosolic and mitotic chromosome fractions, which thereby enables a more precise quantification of enrichment.

An overview of our experimental approach is shown in Fig. 1. In short, demecolcine-trapped mitotic cells are isolated and then incubated under hypotonic conditions to lyse the cells. After removal of the cellular debris by filtration, highly pure mitotic chromosomes and cytosol (containing RNA) are then isolated by centrifugation. An important step in this process is the use of two different filters to remove the cell debris, which leads to a much greater purity of the chromosomal material. Both samples are then further treated to obtain mitotic chromosomes with only the most tightly-bound species and pure samples of the cytosolic material, from which we sequence the mCARs and cytosolic RNA, respectively. A novel aspect of our overall protocol is the quantification of the absolute level of enrichment of the mCARs on the mitotic chromosome relative to that in the cytosol. In particular, with select RNA species, we perform qPCR to determine their extent of chromosomal enrichment, correcting for the differential loss of each fraction during purification. These measurements are then compared with the values obtained from the RNA-seq data, which thereby generates a relationship by which the RNA-seq data can then be generally used to quantify the extent of chromosomal enrichment for all transcripts.

### Cell culture conditions

We examined several different cell lines (Table 1) that were chosen to enable identification of mCAR candidates that are common among (and different between) mammals, specific to epithelial or mesenchymal origin, and different between normal and cancer cells (of different cell-type origin). The A549, HT-1080, STO, and CV-1 cells were all purchased from the Cell Bank/Stem Cell Bank (Chinese Academy of Sciences), while the ARPE-19 cells were purchased from iCell Bioscience Inc. (Shanghai, China). The A549 cells were cultured in RPMI1640 (Gibco, USA); the HT-1080, STO, and CV-1 cells were cultured in DMEM (Gibco, USA); and the ARPE-19 cells were cultured in DMEM/F12 1:1 (Gibco, USA). All cell media were supplemented with 10% FBS (Bovogen, Australia) and 1% penicillin-streptomycin (Gibco, USA). All of the cells were incubated at 37 °C in 5% CO_2_ atmosphere. All cells were tested by MycoBlue Mycoplasma Detector (Vazyme, China) to confirm a lack of mycoplasma contamination (Fig. S1 in figshare^16^). Authentication of the human cell lines was performed using the STR validation (Genetic Testing Biotechnology Corporation, Suzhou, China) (Fig. S1 in figshare^16^).

**Table 1:** Summary of the cell lines used in this work.

Species	Cell line	Tissue and cell type
**Human**	ARPE-19	Retinal pigment epithelium
	A549	Adenocarcinoma, alveolar basal epithelium
	HT-1080	Fibrosarcoma
**Monkey**	CV-1	Kidney, fibroblast
**Mouse**	STO	Embryo, fibroblast

### Mitotic cell synchronization and collection

The cells were cultured until they reached approximately 80% confluency. Subsequently, the culture medium was replaced with fresh medium, and demecolcine (D1925, Sigma-Aldrich, USA) was added to achieve a final concentration of 100 ng/ml. The cells were treated under this condition for 12 h, followed by washing with phosphate-buffered saline (PBS). Mitotic cells were shaken-off from the culture dish, collected and then pelleted by centrifugation at 200 × g for 5 min at 4 °C.

### Validation of the purity of the mitotic cells by FACS

Fluorescence-activated cell sorting (FACS) was used to determine the proportion of mitotic cells in the sample following a published protocol with minor modifications^18^. The cells were fixed in 70% ethanol at 4 °C and resuspended in 500 μl PBS after incubation with RNase A (0.2 μg/μl) and Triton X-100 (0.1% W/V) at 37 °C. PI (P4864, Sigma-Aldrich, Germany) was then added (20 μg/ml) for DNA staining. The cells were then analysed using the BD FACS LSRFortessa flow cytometer (BD Bioscience, USA). The FACS data were analysed using the ModFit32 software^19^, which confirmed a high purity of mitotic cells in these samples (Fig. S2 in figshare^16^).

### Purification of mitotic chromosomes and the corresponding cytosolic fraction

**Fig. 2:**
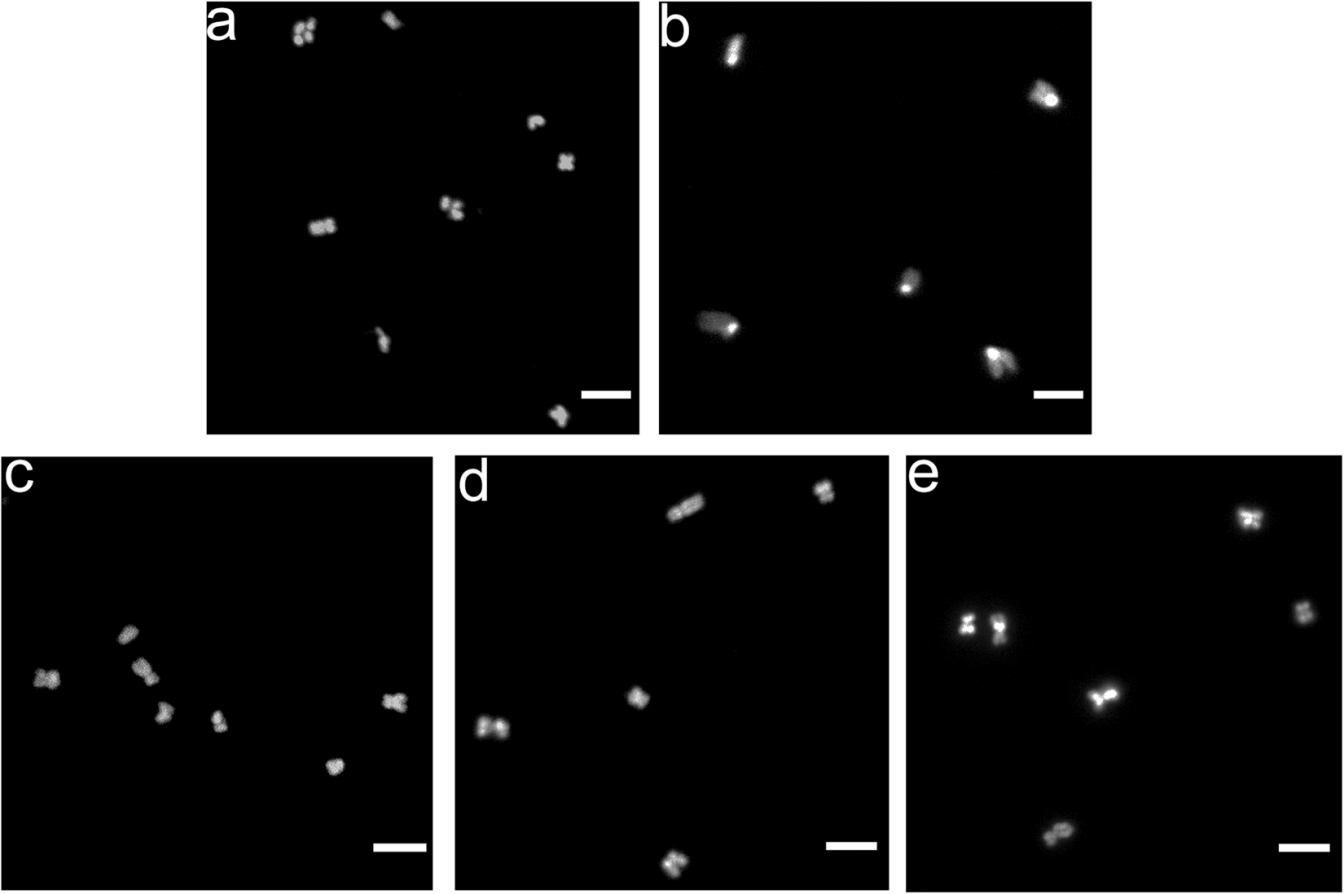
**Assessment of the purity and morphology of the isolated mitotic chromosomes**. **(a–e)** Confocal fluorescence microscopy images of DAPI-stained purified mitotic chromosomes of the **(a)** monkey cell line, CV-1; **(b)** mouse cell line, STO; and human cell lines **(c)** A549, **(d)** HT-1080, and **(e)** ARPE-19. Each sample exhibits the expected overall structure of these chromosomes, including the distinct acrocentric chromosomal architecture of the mouse chromosomes and the conventional “X”-shaped structures of the monkey and human chromosomes. Scale bar: 5 µm.

Mitotic chromosomes were purified following a published method^14,20^ that was modified to substantially increase both the purity and final yield with a shortened purification process. Briefly, the cells were first incubated in a hypotonic solution (75 mM KCl) followed by centrifugation at 1,750 × g at 4 °C. The cells were then resuspended with pre-chilled (4 °C) polyamine (PA) buffer (15 mM Tris-HCl, 0.2 mM spermine, 0.5 mM spermidine, 0.5 mM EGTA, 2 mM EDTA, 80 mM KCl, 20 mM NaCl, 0.1 mM PMSF, 1 mg/ml digitonin) which has been shown to protect the mitotic chromosome morphology^21^, and then incubated for 10 min on ice. Mitotic chromosomes were then released from the cells with a homogenizer. The resulting homogenate was centrifuged at 190 × g for 5 min at 4 °C and the supernatant (containing released mitotic chromosomes) was collected in a new tube. The pellet was then resuspended and additional homogenization was performed to rupture any remaining intact cells. After centrifugation at 190 × g for 3 min, the supernatant was combined with the previously collected supernatant and the combined supernatant was filtered twice through first a 10 μm filter (NY41002500, Millipore, UK) and then a 5 μm filter (SVLP01300, Millipore, UK) with a syringe pump (LSP02-1B, LongerPump, China). The resulting filtrate was then centrifuged at 1,750 × g for 10 min at 4 °C to separate the chromosomes (pellet) from the (crude) cytosolic material (supernatant). To remove small debris from the cytosolic material, the supernatant was collected and centrifuged at 10,000 × g for 5 min twice. Finally, 1 ml of the supernatant fraction was collected and 1 ml of Trizol reagent (Invitrogen, US) was added to denature cellular material in preparation for RNA extraction. For the chromosomes, the pellet was resuspended in 3 ml of PA buffer supplemented with 0.2 M NaCl (high salt buffer) and incubated for 25 min on ice to remove loosely bound materials^14^. The resuspended mitotic chromosomes in the high salt buffer were then centrifuged at 1,750 × g for 6 min at 4 °C, and the resulting pellet was resuspended in 500 μl of PA buffer without digitonin. The morphology of the chromosomes was examined by fluorescence microscopy. For this, the mitotic chromosome sample was deposited on a glass slide using Cytospin 4 (Thermo Fisher Scientific, USA) at 1,000 rpm for 5 min, followed by incubation with DAPI (H-1200, Vector, USA). The chromosomes were then imaged using confocal microscopy (A1Si, Nikon, Japan). Figure 2 shows that the mitotic chromosomes after this somewhat extensive procedure retain their characteristic structures. A fraction of the purified chromosomes was concentrated with centrifugation at 10,000 × g and then cryopreserved at -20 °C for subsequent DNA extraction (for quantification, see below). The remaining mitotic chromosomes were treated with Trizol in preparation for RNA extraction.

### RNA purification

Extraction of both cytosolic RNA and mitotic chromosome-associated RNA were carried out using Trizol in conjunction with the Phasemaker tube system (Thermo Fisher Scientific, USA). The protocol was performed according to the manufacturer’s instructions with slight modifications. In particular, we followed these instructions except that, during the incubation with isopropanol, we added a combination of 1/10 volume of 3 M sodium acetate (NaAc, pH 5.5), 1 μl of glycogen (20 mg/ml), and an equal volume of isopropanol, which was then incubated at -30 °C for more than 2 h. In addition, we performed an additional step to remove any DNA within the final pellet by resuspending in DEPC-treated water with DNase I (0.5 U/µl, NEB, USA) and RiboLock RNase inhibitor (0.8 U/µl, Thermo Fisher Scientific, USA). The RNA was finally purified using phenol-chloroform-isoamyl alcohol (25:24:1) extraction and ethanol precipitation. The precipitated RNA was dissolved in DEPC-treated water for future use. The RNA concentration was determined using a Qubit 3.0 Fluorometer (Thermo Fisher Scientific, USA) with the Qubit RNA HS Assay Kit (Thermo Fisher Scientific, USA).

### RNA-seq library preparation and sequencing

About 200 ng of RNA for each sample was used to prepare libraries using the KAPA RNA HyperPrep Kit with RiboErase (which removes rRNA) (KK8560, Roche/KAPA Biosystems, South Africa), with some modifications. In particular, we followed the instructions with the kit except that we used a 3x bead-based clean-up method to purify the RNA and a 1x bead-based clean-up method to purify the DNA with adapter sequences. This combined purification strategy ensured the recovery of insert fragments longer than 100 bp to maximize the collection of snoRNA and other similarly-sized ncRNA insert fragments. The concentration of these libraries was determined using a Qubit 3.0 Fluorometer with the Qubit DNA HS Assay Kit (Thermo Fisher Scientific, USA). The samples were paired-end sequenced with Illumina Nova-seq.

### Data analysis

We obtained 3 replicates of each of the mCAR and cytosolic RNA samples for each cell line. Overall, we obtained about 2,133 M raw reads pairs (2 × 150 bp) from these 30 libraries (Table 2). Cutadapt-3.5^22^ (with parameters of -max-n 0--minimum-length 100) was used to remove sequencing adapters from the raw reads and Trimmomatic-0.36^23^ (with parameters of PE SLIDINGWINDOW:3:10 LEADING:10 TRAILING:10 MINLEN:100) was used to remove low-quality reads. Residual ribosomal RNAs were removed by SortMeRNA-v2.1b^24^ in the pair-end mode with default parameters. The Q30 profiles of the cleaned reads generated by FastQC-v0.11.5^25^ were manually inspected to ensure sufficiently good data quality for further analysis. After this, the cleaned reads were mapped to the reference genome using hisat2-2.0.5 in a strand-specific mode (with parameters of--rna-strandness FR)^26^ using the human reference genome GRCh38, the green monkey reference genome ChlSab1.1, or the mouse reference genome GRCm38. In the end, we obtained, overall, 1,410 M mapped read pairs (~47 M for each replicate on average).

**Table 2:** Basic statistics of the RNA-seq data.

Sample	Raw read pairs	Read pairs after low quality reads removal	Clean read pairs after rRNA removal	Mapped read pairs	Uniquely mapped read pairs
Total	2,132,594,270	1,815,613,968	1,730,665,525	1,409,552,973	1,171,968,890
A549_Cyto1	48,611,359	35,778,329	34,765,749	30,593,767	28,627,993
A549_Cyto2	56,934,404	43,477,588	42,203,879	36,376,524	33,890,854
A549_Cyto3	55,628,870	42,562,442	41,369,026	35,925,149	33,151,781
A549_mCARs1	59,070,144	42,197,746	40,288,077	35,054,176	26,443,565
A549_mCARs2	49,208,578	42,167,965	40,362,368	34,426,681	25,477,983
A549_mCARs3	54,681,667	42,953,750	41,410,043	36,052,879	28,775,174
ARPE-19_Cyto1	68,764,637	60,084,642	58,662,714	51,647,133	45,366,013
ARPE-19_Cyto2	69,454,536	60,685,866	58,935,141	51,917,058	46,686,731
ARPE-19_Cyto3	83,871,698	70,535,881	68,627,465	60,309,434	53,124,566
ARPE-19_mCARs1	79,187,101	64,614,580	61,008,235	52,463,857	32,702,724
ARPE-19_mCARs2	68,417,146	59,290,343	55,253,349	44,957,112	27,196,041
ARPE-19_mCARs3	76,180,088	67,018,346	62,632,177	53,039,777	32,983,150
HT-1080_Cyto1	87,268,380	77,704,801	75,836,665	66,660,474	60,539,794
HT-1080_Cyto2	81,990,820	71,058,630	69,283,157	59,758,459	52,987,877
HT-1080_Cyto3	71,892,338	59,803,442	58,482,738	51,053,202	45,153,256
HT-1080_mCARs1	93,155,010	84,522,136	80,084,442	65,437,613	47,996,785
HT-1080_mCARs2	82,728,047	73,177,744	68,893,658	56,636,765	39,330,547
HT-1080_mCARs3	69,222,533	61,714,964	58,436,223	49,412,756	34,124,117
CV-1_Cyto1	71,799,660	63,093,820	61,019,817	48,758,095	41,593,455
CV-1_Cyto2	71,631,777	63,876,311	62,033,011	49,866,436	42,775,646
CV-1_Cyto3	70,393,871	62,496,403	60,615,322	48,597,136	41,877,712
CV-1_mCARs1	73,241,572	64,555,663	62,957,956	38,586,667	36,717,970
CV-1_mCARs2	70,035,908	64,230,101	62,578,001	41,184,041	39,164,523
CV-1_mCARs3	70,721,110	64,080,029	62,444,049	43,383,788	41,281,787
STO_Cyto1	75,820,482	62,979,141	54,629,887	45,002,531	37,465,080
STO_Cyto2	68,874,054	56,534,056	49,133,985	40,064,451	32,920,909
STO_Cyto3	69,221,350	58,845,914	51,654,037	42,952,155	35,767,625
STO_mCARs1	86,423,565	72,159,399	69,070,001	51,225,334	46,832,098
STO_mCARs2	76,586,107	66,571,529	63,733,674	48,323,246	44,481,919
STO_mCARs3	71,577,458	56,842,407	54,260,679	39,886,277	36,531,215

**Fig. 3:**
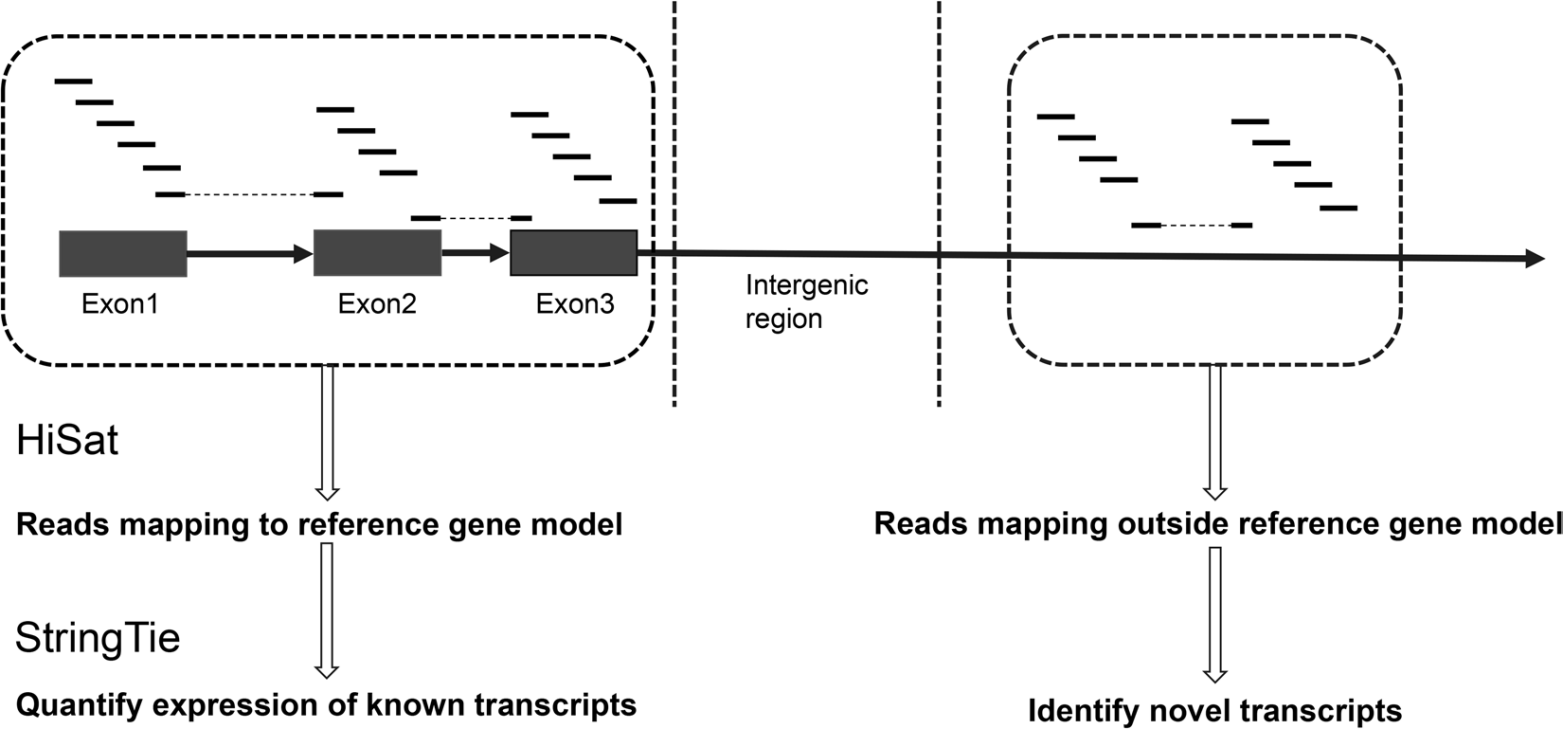
**Overview of the bioinformatic mapping process**. The annotated RNAs were first identified by mapping the reads to the reference genome using HiSat and StringTie, after which the novel mCARs were *de novo* assembled with the remaining reads that mapped 2 kb upstream of a TSS or 2 kb downstream of a TTS.

The level of expression of both transcripts and genes were calculated with StringTie-1.3.3^27^ (with parameters of -e -b) based on the reference gene models from the Ensembl database (Homo_sapiens.GRCh38.105.chr.gtf for human; Mus_musculus.GRCm38.102.chr.gtf for mouse; and Chlorocebus_sabaeus.ChlSab1.1.105.chr.gtf for green monkey). Uniquely mapped clean read pairs which were compatible with the reference gene model were used to quantify the expression level of the annotated transcripts (including transcript isoforms) (Fig. 3), retaining those transcripts at TPM (Transcripts Per Million) > 1 for further analysis. For our analysis of novel transcripts, we excluded presently annotated gene bodies and proximal loci to minimize possibly mis-identifying a transcript as novel. Specifically, read pairs that mapped 2 kb upstream of a TSS (transcription start site) or 2 kb downstream of a TTS (transcription termination site) were further analysed with an additional StringTie assembly step (with parameters of -b; without -e) for the assembly of the novel transcripts (Fig. 3). For this analysis, we only used the reads associated with the chromosome (TPM > 5) (and not the cytosolic RNA) to enable identification of novel mCARs in particular. Since the identification of a novel RNA transcript relies on *de novo* assembly (unlike the identification of annotated transcripts that relies on mapping), we considered only the transcripts assembled in this second step with TPM > 5 as novel mCARs^28^.

### Quantification of the mitotic enrichment of the mCARs

When aiming to quantify the extent to which the mCARs are enriched on the chromosomes relative to the cytosol, there are two experimental issues that must be addressed. First, there is a significant loss of the chromosomes during the purification process and a far smaller degree of loss of the cytosolic material during its purification. Thus, simply comparing the quantity of transcripts in the two fractions without accounting for this differential loss during sample purification will substantially mis-characterize the level of enrichment. Ideally, it would be best to compare the RNA copy numbers of the two fractions from the same number of cells. Second, owing to significant differences in the composition and number of the RNAs in the chromosome-associated fraction and the cytosolic fraction (even if they are determined from an equal number of cells), simply relying on the TPM values in the RNA-seq data to determine mitotic enrichment can lead to significant errors^29,30^. There are available computational methods (such as TMM^31^) that are frequently used to normalize reads between different samples that could have been employed to overcome these problems. These methods estimate a factor for normalization based on the assumption that the majority of genes, common to both samples, are not differentially expressed^29,31^. However, a priori, there is no reason to expect any specific degree of common expression (high or low) between the cytosolic and mitotic chromosome-associated transcripts. Therefore, instead, we experimentally determined the appropriate normalization factor, adjusting the number of transcripts obtained in the two fractions (cytosolic or chromosome-associated) to that as if they were obtained from the same number of cells, using the amount of genomic DNA in each fraction as a proxy for cell number.

In particular, we first experimentally determined a conversion factor that relates the TPM mCAR/cytosolic RNA fold-change ratio of several select genes to that measured using qPCR from the same samples. As shown in Fig. S3 in figshare^16^, the TPM ratio for these genes is indeed linearly proportional to the qPCR ratios over a broad range of fold-change values. We then used the slopes of these plots to convert all of the TPM mCAR/cytosolic RNA ratios to their qPCR fold-change counterparts for each cell type (Table S3 in figshare^16^). For the RT-qPCR, each sample was subjected to synthesis of the first-strand cDNA using the Superscript SSIV reverse transcriptase (Invitrogen, USA) according to the manufacturer’s protocol, starting with 40 ng of total RNA (for both mCAR and cytosolic RNA samples). An appropriate quantity of the resulting cDNA was used as the template, and a 10 μl qPCR reaction system was prepared using ABI PowerUp MIX (Invitrogen, USA). The amplification and detection of the target sequences were carried out using the QuantStudio™ 3 Real-Time PCR instrument (ABI, USA). The primers of target RNAs for qPCR are shown in (Table S4 in figshare^16^).

We next adjusted these ratios to account for differential loss. In short, we used the amount of genomic DNA that is present after the purification procedures as a proxy measure of the cell number and adjusted these ratios by the factor by which the measurements of the genomic DNA of the two fractions differed. At the beginning of the entire procedure (Fig. 1), we first determined the number of cells in a given sample using a hemocytometer counting chamber (Green and Sambrook 2019). After aliquoting ~10^7^ cells of the sample for the isolation of the mCARs and the cytosolic RNA, we used the remaining portion of the sample to obtain a DNA calibration curve that relates the cell number to the quantity of genomic DNA measured. We quantified the DNA content using a Qubit 3.0 Fluorometer (Thermo Fisher Scientific, USA) instrument and the Qubit DNA HS Assay Kit (Thermo Fisher Scientific, USA). We then measured the amount of genomic DNA in the mitotic chromosome fraction after purification and used the DNA calibration curve to determine the number of cells associated with this DNA content. The fraction of cells that was (effectively) retained during purification calculated in this way for each cell-type is shown in Table S3 in figshare^16^. We assumed that the cell number that is associated with the amount of cytosolic RNA is that of the input cells (namely, 10^7^ cells). Thus, in the end, the extent of enrichment of each species is given as if both mCAR and cytosolic RNA were obtained from the same number of cells.

## Data Records

The FASTQ files for the raw data have been deposited in the NCBI Sequence Read Archive (SRA)^32^ under accession SRP479011. Additional data analysis and qPCR primer information (Tables S1-S4) and figures (Fig. S1-S8) have been deposited in figshare^16^.

## Technical Validation

### Evaluation of RNA, library, and data quality

**Fig. 4:**
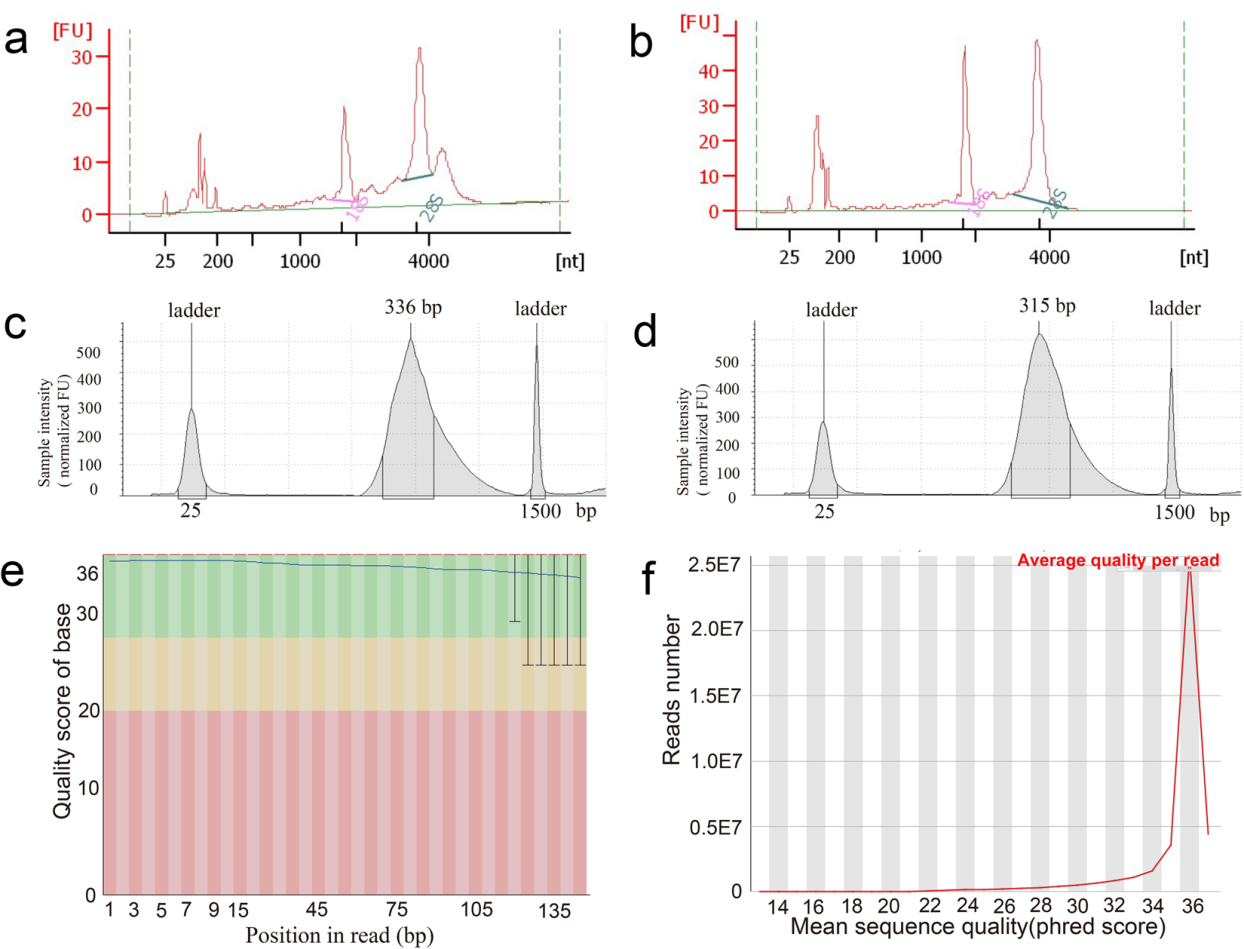
**Quality of the purified RNAs, sequencing libraries, and sequencing reads**. **(a,b)** Electropherogram of representative **(a)** mCARs and **(b)** cytosolic RNA obtained with the A549 cell. **(c,d)** Electropherogram of a representative sequencing library from **(c)** mCARs and **(d)** cytosolic RNA for the A549 cell. **(e)** Distribution of quality scores by base pair for a representative FASTQ file from RNA-seq data for the A549 cell. The quality scores are defined as -10log10(P), where P is the probability that a base call is erroneous. In this image, the background colors reflect very good quality calls (green, quality scores: 28-40), calls of reasonable quality (orange, quality scores: 20-28), and calls of poor quality (red, quality scores: 0-20). The blue line in the graph represents the mean quality scores. **(f)** Distribution of the mean quality score by reads for a representative FASTQ file from RNA-seq data in the A549 cell. The phred scores are also defined as -10log10(P), where P is the probability that a base call is erroneous.

The quality of the RNA from both mCAR and cytosolic RNA samples was analysed with the total RNA 6000 Pico Kit (Agilent, Germany) using an Agilent 2100 Bioanalyzer with RNA 6000 Pico Lab Chip (Agilent Technologies, USA). Measurements for a sample from the A549 cells are shown in Fig. 4a,b, revealing that the RNA is of high purity and quality. Other cells exhibited a similar degree of good quality (Fig. S4 in figshare^16^). The libraries were analysed with the Agilent 2200 Bioanalyzer with High Sensitivity D1000 Reagents (Agilent Technologies, USA). This showed that the libraries indeed have good quality with sharp peak in DNA size at about 330 bp (Fig. 4c,d for the A549 cells and Fig. S5 in figshare^16^ for the other cells). After sequencing, the data quality of the sequencing (namely, base quality score and reads quality score) was validated by FastQC^25^, which also showed a high quality of this data (Fig. 4e,f for the A549 cells and Fig. S6 in figshare^16^ for the other cells).

### Evaluation of reproducibility between independent biological replicates

**Fig. 5:**
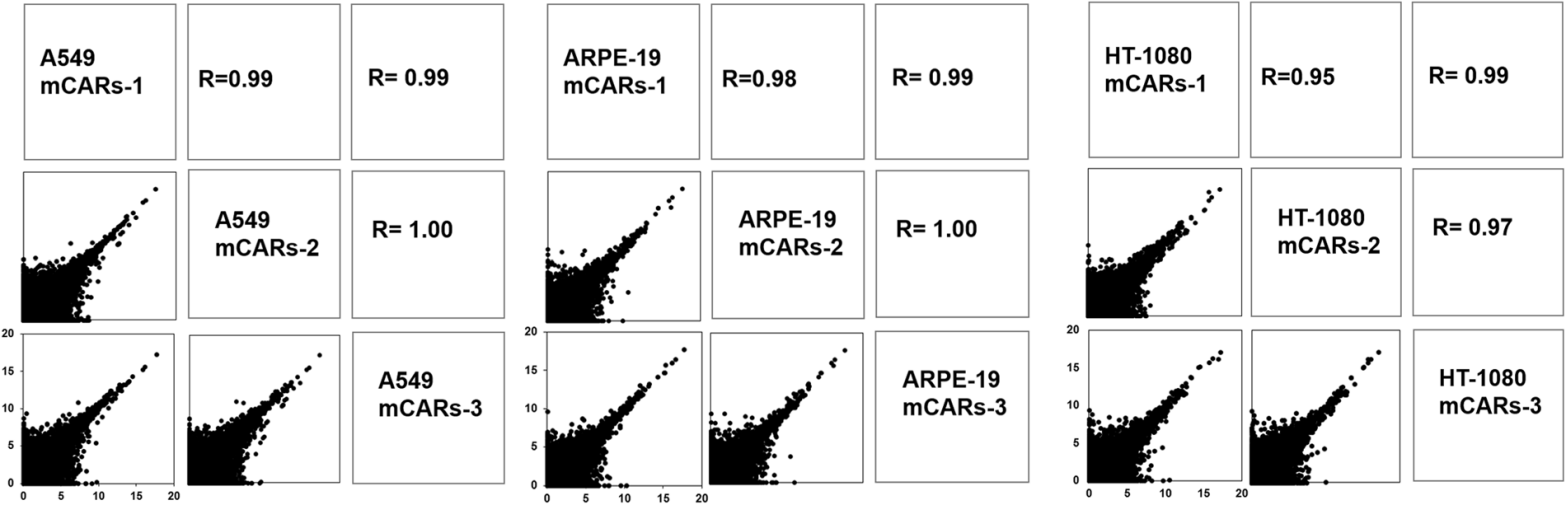
**Pairwise scatter plots between independent biological replicates of mCARs of the A549, ARPE-19, and HT-1080 Cells**. Shown are the Pearson correlation (R) coefficients measured from pairwise comparisons between the samples. Each dot represents one transcript, with the plots showing log2(TPM + 1) for each transcript.

To validate the reproducibility of the three independent biological replicates in each sample, we examined the extent to which the replicates were correlated. As shown in Fig. 5 and Fig. S7 in figshare^16^, there is indeed a high degree of reproducibility between the mCAR samples. Similar results were observed with the cytosolic RNAs (Fig. S8 in figshare^16^).

### Evaluation of mCARs with published data

As an additional means of validation of the mCARs identified here, we compared the mCARs that we identified from the A549 cells with those obtained by Shen *et al*.^15^ from the same cell type. As the latter described their mCARs in terms of genes, rather than transcript isoforms, this comparison was performed at the gene level. We found that over 4,115 of the annotated mCAR genes in our list (95%) were also present in the genes identified in Shen. *et al*., and thus are highly consistent with this data.

## Data Availability

No custom code was used in the analysis of the data in this manuscript.
